# Localization and Mapping on Agriculture Based on Point-Feature Extraction and Semiplanes Segmentation From 3D LiDAR Data

**DOI:** 10.3389/frobt.2022.832165

**Published:** 2022-01-28

**Authors:** André Silva Aguiar, Filipe Neves dos Santos, Héber Sobreira, José Boaventura-Cunha, Armando Jorge Sousa

**Affiliations:** ^1^ INESC TEC—INESC Technology and Science, Porto, Portugal; ^2^ School of Science and Technology, University of Trás-os-Montes e Alto Douro, Vila Real, Portugal; ^3^ FEUP, University of Porto, Porto, Portugal

**Keywords:** localization, mapping, 3D LIDAR, semiplanes, agriculture

## Abstract

Developing ground robots for agriculture is a demanding task. Robots should be capable of performing tasks like spraying, harvesting, or monitoring. However, the absence of structure in the agricultural scenes challenges the implementation of localization and mapping algorithms. Thus, the research and development of localization techniques are essential to boost agricultural robotics. To address this issue, we propose an algorithm called VineSLAM suitable for localization and mapping in agriculture. This approach uses both point- and semiplane-features extracted from 3D LiDAR data to map the environment and localize the robot using a novel Particle Filter that considers both feature modalities. The numeric stability of the algorithm was tested using simulated data. The proposed methodology proved to be suitable to localize a robot using only three orthogonal semiplanes. Moreover, the entire VineSLAM pipeline was compared against a state-of-the-art approach considering three real-world experiments in a woody-crop vineyard. Results show that our approach can localize the robot with precision even in long and symmetric vineyard corridors outperforming the state-of-the-art algorithm in this context.

## 1 Introduction

The development of autonomous robots in agriculture is a challenging and active research topic (Emmi et al., 2014). To implement such systems, the autonomous navigation issue must be solved, i.e., robots should be capable of driving autonomously within multiple environments Shalal et al. (2013). Consequently, autonomous robotic platforms should be endowed with robust localization systems, that allow recovering their absolute pose in the agricultural environment (Vougioukas, 2019). Simultaneous Localization and Mapping (SLAM) allows calculating the travelled trajectory while mapping the environment simultaneously (Bailey and Durrant-Whyte, 2006; Durrant-Whyte and Bailey, 2006). In agriculture, the implementation of SLAM is particularly important since it leads to creating maps that farmers can use in various tasks. When robots have this ability, they can perform several autonomous operations such as precision agriculture (application of fertilizers, nutrients and water), plant protection, harvesting, monitoring, and planting (Bergerman et al., 2016; Roldán et al., 2018; Pinto de Aguiar et al., 2020). Even so, the implementation of SLAM in outdoor agricultural environments can be challenging since the characteristics of illumination and terrain irregularities can difficult the perception stages, and therefore, compromise the SLAM systems (Aguiar et al., 2020a).

To perform accurately, one of the most important steps of SLAM is perception. In this context, the use of 3D LiDARs has become popular in outdoor environments since they allow a high-range field of view perception of the environment. The point clouds generated by these sensors contain several types of features that are important for SLAM approaches. Regarding point features, one important descriptor is *smoothness* (Zhang and Singh, 2017), that for a given point ***p***_***i***_ and a set of continuous points *S*, can be calculated as

$$c=\frac{1}{\left|S|\cdot \|p_{i}\right\|}\left|\left|\underset{j\in S,j\neq i}{\sum }\left(p_{j}-p_{i}\right)\right|\right|.$$ (1)

With the application of threshold levels to *c*, two main types of point features can be extracted. Features with small values of *c* are present in high-curvature locations and therefore are called edge features. On the contrary, features with high smoothness are called planar features. Shan and Englot (2018) use these concepts in their proposed SLAM pipeline, LeGO-LOAM. In this work, the raw point cloud is first segmented to remove noise, and then edge and planar features are extracted. Other approaches propose the extraction of point features in the SLAM context. Steder et al. (2010) project the point cloud onto a range image and calculate the second derivative of the depth. With this formulation, high-curvature features are extracted from the range image. Chen et al. (2015) extract curb features from a 3D point cloud with a range up to 50 meters. This approach uses a distance criteria, and a Hough transform to process the point cloud and collect the desired features.

Point features extracted from 3D point clouds can have high computational cost in localization and mapping algorithms due to their usual high density. Even if the SLAM approaches are robust to this issue, the fusion of these perception techniques with other feature types can improve performance (Grant et al., 2019). Thus, as detailed in Table 1, many works use plane features in the mapping and localization stages. To extract such features, one of the most common techniques is Random Sample Consensus (RANSAC) (Gee et al., 2008; Ulas and Temeltas, 2012; Taguchi et al., 2013; Elghor et al., 2015). This algorithm receives as input a set of points and calculates the best fitting plane to those points, removing the input set’s outliers. Other approaches use Convolutional Neural Networks (CNN)s (Yang et al., 2016) or Principal Component Analysis (PCA) (Viejo and Cazorla, 2007) for plane extraction. In terms of representation, a plane *i* is usually characterized as

$$m_{\gamma_{i}}=\left\{\pi ,\;d\right\},$$ (2)

where $\pi ={\left[\pi_{1},\pi_{2},\pi_{3}\right]}^{T}$ represents the plane unit normal vector, and *d* the plane distance to the origin. Other representations are also present in the literature. For example, Elghor et al. (2015) also preserve the number of inliers found in the RANSAC procedure in the plane representation. Also, Gee et al. (2008) represent a plane by its origin and two orthogonal basis vectors. All these representations are suitable for infinite planes. For localization and mapping, this representation can be improved using semiplanes. With this type of features, the matching procedure becomes more robust since other correspondence techniques can be applied, such as semiplane overlapping (Yang et al., 2016). Ulas and Temeltas (2012) use a Convex Hull algorithm to extract semiplanes extremas and feed an outdoor SLAM algorithm. Similarly, Weingarten and Siegwart (2006) represent semiplanes by a set of convex polygons and use them in a 3D SLAM algorithm. Lenac et al. (2017) also use this representation and incorporate the polygons extremas in each semiplane characterization vector.

**TABLE 1:** Summary of the current state-of-the-art on plane-based localization and mapping.

References	Application	Feature extraction	Mapping
Taguchi et al. (2013)	3D reconstruction of indoor spaces using hand-held sensors.	Points: image feature detector; planes: RANSAC algorithm.	RANSAC-based registration algorithm.
Yang et al. (2016)	Localization and mapping of low-texture indoor environments.	CNN-based plane detection.	Point and semiplane registration.
Viejo and Cazorla (2007).	Egomotion estimation in indoor and outdoor semi-structured environments.	Plane extraction using a PCA technique.	Iterative Closest Point (ICP) algorithm used for plane registration.
Grant et al. (2019)	Velodyne point-plane SLAM in challenging indoor and outdoor environments.	Find groups of points that arise from planar surfaces in a scan-line basis (Grant et al., 2013).	Registration with a developed algorithm: Iterative Closest Point Plus Plane Optimization (IC3PO).
Zhang et al. (2019b)	SLAM in indoor environments.	Plane segmentation using a connected component-based approach.	Points added by triangulation and observed planes added if no correspondence is found.
Kaess (2015)	Mapping of indoor environments using hand-held sensors.	Plane segmentation from point cloud data.	Infinite plane representation and mapping.
Weingarten and Siegwart (2006)	Localization and mapping of indoor environments.	Divide and conquer approach: best-fitting planes from small regions (Weingarten et al., 2003).	3D map builds using an Extended Kalman Filter (EKF).
Gee et al. (2008)	SLAM in indoor environments using hand-held sensors.	Planar surfaces extracted using RANSAC.	Registering based on similarity test.
Elghor et al. (2015)	3D reconstruction of indoor environments.	Planes extracted using RANSAC.	Planes registered and fused using a weight function.
Lenac et al. (2017)	Planar representation of indoor and outdoor environments.	Plane segments extracted by a 2D Delaunay triangulation.	Registration using the overlapping between planes.
Ulas and Temeltas (2012)	Outdoor SLAM.	Planes extracted using RANSAC.	Planes matched and registered using: orientation, translation and closeness.
Our approach	Autonomous navigation in outdoor agricultural environments.	Point-wise and three stage semiplane-wise feature extraction.	Point registration and semiplane matching and merging algorithm for registering and mapping.

Plane mapping and registration can be more challenging than feature point mapping. In the latter, the general approach is to use a nearest neighbor search, optimized by efficient data structures such as 3D voxel maps. Two main factors are usually considered to solve plane matching and mapping: the difference between the plane normals and plane-to-plane distance (Viejo and Cazorla, 2007; Grant et al., 2013). With the consideration of bounded planes, Pop-up SLAM (Yang et al., 2016) also uses the overlapping area between semiplanes. After a successful matching procedure, the plane-based mapping step either adds new features to the map or updates the existing ones in case of correspondence. In SLAM, the mapping procedure is interdependent of localization. From Table 1, one can verify that most works use graph-based optimization to localize the robot using planes. For example, Zhang X. et al. (2019) build a pose graph where points and planes are marked as landmark nodes, and add structural constraints between planes in the graph. Kaess (2015) formulates planar SLAM as a factor graph finding a solution for the localization and mapping through least-squares optimization. Some works also adapt the ICP scan-matching algorithm to be used considering planar features. Viejo and Cazorla (2007) propose a two-step ICP that considers separate orientation and position alignment. This approach uses information given by the normal vector orientation and the geometric plane position. In addition, some works are based on Gaussian filters (Gee et al., 2008; Ulas and Temeltas, 2012; Zhang X. et al., 2019). In these, the state vector comprises the robot pose and the plane landmarks. In comparison with point feature-based SLAM, this approach has the advantage of reducing the state’s dimension.

Given all of the above, it is clear that 3D LiDARs provide rich information for localization and mapping approaches. Besides the previously mentioned LeGO-LOAM approach, and its ancestor LOAM (Zhang and Singh, 2014), other approaches use this sensor to provide reliable SLAM systems. Kuramachi et al. (2015) propose a range and inertial odometry algorithm that fuses a 3D LiDAR and a gyroscope to recover the 6-DoF robot pose and map the environment. This LiDAR odom etry technique can either be approached traditionally using iterative algorithms, or in more sophisticated manners, such as using Artificial Intelligence (AI). For example, Choy et al. (2020) propose a deep global registration algorithm that is designed for pairwise registration of 3D scans. The key innovation of this approach is the use of a 6-DoF Convolutional Neural Network (CNN) for correspondence confidence prediction. In the same context, Li and Wang (2020) propose DMLO, a deep matching LidAR odometry algorithm which presents a learning-based matching network which provides accurate correspondences between two scans. In this work, we make use of a rich feature extraction process that considers both point and semiplane features, and implement a filter-based algorithm for localization and mapping. As represented on Table 1, to the best of our knowledge, the Particle Filter (PF) has not been approached together with semiplanes features in the SLAM context. The most common approach is to use optimization-based localization algorithms such as Bundle Adjustment and factor graph optimization. Thus, in this work, we extend the state-of-the-art to consider the use of a 6-DoF PF that supports two modalities of features: point-wise and semiplane-wise features. This filter-based algorithm was initially applied to robotics to solve the localization and kidnapping problems (Thrun, 2002). Zhang Q.-B. et al. (2019) use the PF to develop a robust localization algorithm that works when a priori environment map is available, considering range observations. Due to its capacity to accommodate multi-dimensional problems, the PF was later used to solve the SLAM issue. FAST-SLAM (Montemerlo et al., 2002) is one of the most popular examples, being able to solve the SLAM problem with a PF considering a landmark-based feature extraction procedure. In the agricultural context, the PF was also applied in autonomous navigation algorithms. Hiremath et al. (2014) use a PF to implement a row following algorithm in a maize field. The filter state is composed of robot heading, lateral deviation, distance between the rows of plants and the end of the rows. Similarly, Blok et al. (2019) use the PF and a 2D laser sensor to localize an agricultural platform for in-row navigation in orchards.

In this work, we propose VineSLAM (dos Santos et al., 2016; Aguiar et al., 2021), a 6-DoF SLAM algorithm for agricultural environments that uses point and semiplane features extracted from 3D point clouds. Our approach presents the following main contributions:

• A three-stage algorithm for semiplane extraction;
• A semiplane matching and merging algorithm that allows efficient registering and mapping;
• A novel localization procedure based on a PF that can use both point and planar information.

We model the agricultural environment as points and semiplanes. In each time step, edge and planar point features are extracted, and three semiplanes are searched in the environment. The first semiplane is the ground, and the other are two semiplanes, one in each side of the robot, that present the higher number of inlier points. This formulation is a reaction to the context where VineSLAM is intended to solve localization and mapping: agricultural cultures mainly characterized by woody-crop topologies. Thus, besides edge and planar features, usually only three semiplanes are available in the environment. These semiplanes are essential to the estimation of the three components of the robot’s orientation. The ground plane is particularly important to estimate the roll and pitch components, and the vegetation planes to estimate the yaw component. Without these features, the algorithm would rely only on point-based features which could lead to drift in the orientation components over time. Also, if we rely only on semiplanes, we would always have to extract a minimum of three non-coplanar semiplanes to compute the 6-DoF localization of the robot. Thus, it is essential to merge the point and planar features.

Our approach relates to the state-of-the-art feature extraction and matching steps but differs in the registration. We aplly a plane merging algorithm that constantly updates and grows the semiplanes present on the map. With this approach, we can capture large planar ground surfaces, as well as extensive vegetation planes. Also, we propose a novel localization algorithm that uses a PF fusing information at both point and planar levels. As reported in Table 1, most works in the state-of-the-art apply plane-based localization and mapping in indoor environments. Our approach is suitable for unstructured outdoor environments, and it was tested in a woody-crop vineyard. The PF approach was adopted in this work due to three main reasons. The first is relative to sensor fusion. Our aim is to implement a generic algorithm that is agnostic to the type of sensors used, i.e., an approach that can use any kind of sensor if an adequate weight function is provided for each one of them. With this, particles should be weighted by the combination of all the weight sub-functions. The second motivation for the use of this kind of filter to solve the SLAM problem is the straightforward parallelization scheme that it provides. Powerful processors such as Graphics Processing Units (GPU)s can be used considering that the calculations performed per particle can be executed in a separate processing core. Finally, the third is the support for multiple noise distributions in relation with other approaches that usually only support Gaussian noise. In this way, particles can be sampled and innovated with the distribution that best fits with the robot itself, and the scenario where it is inserted in.

The remainder of this paper is structure as follows. Section 2 details the contributions of this work. Section 3 contains two simulation experiences to validate the proposed approach. Section 4 presents the test and validation of this work in real-world experiments. Finally, Section 5 details the conclusions of this work.

### 2 VineSLAM: Localization and Mapping on Agriculture

**FIGURE 1:**
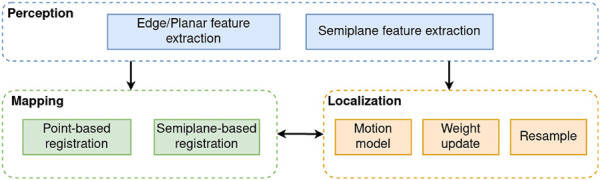
System architecture partitioned in three main layers: perception, localization and mapping.

This work proposes VineSLAM, a localization and mapping algorithm based on 3D points and semiplane features extracted from an input point cloud. Figure 1 shows a high-level representation of the approach.

The system is divided in three main layers:

• Perception: 3D point cloud processing to extract edge, planar and semiplane features;
• Mapping: Multimodal registration of the types of features extracted to build a consistent 3D map of the agriculture environment;
• Localization: PF-based procedure that uses both point- and semiplane-based information to localize the robot.

Thus, our approach is able to efficiently extract point and semiplane features from a 3D point cloud, and use them to build a map of the crop and localize the robot within this map.

### 2.1 Perception

In the perception stage, three feature types of two different modalities are extracted: edge and planar features (points), and semiplanes. The point features are searched in sharp edges and planar surfaces. In the semiplane extraction case, this work searches for three semiplanes in the environment for each frame. The first is a flat ground surface, and the others are two semiplanes, one in each side of the robot. This formulation allows the extraction of large ground surfaces by recurrent plane registration. In woody-crop cultures, these semiplanes usually extract the morphology of the vegetation canopies. The extraction of point and semiplane features is described in 1) and 2) respectively.

**FIGURE 2:**
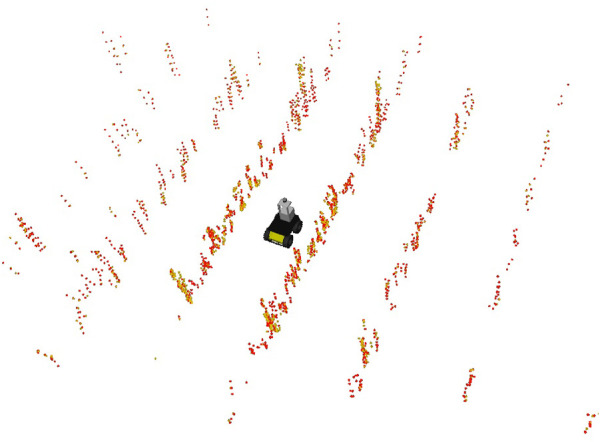
Corner (yellow) and planar (red) feature extraction example in a woody crop vineyard.

1) Point-level feature extraction: To extract point features, our approach relates with LeGO-LOAM Shan and Englot (2018) in that it uses the smoothness descriptor *c* present in (Eq. 1). Points are projected into a range image and sorted by their value of *c*. The points with larger *c* are considered edge features, and the ones with lower *c* are considered planar features. Figure 2 shows an example of this feature extraction procedure in a woody crop vineyard.
2) Semiplane-level feature extraction: Our approach can simultaneously use points and semiplanes to map the agriculture and localize the robot. The representation of semiplanes includes more dimensions than point features, and their extraction involves more complex procedures. In this work, we represent semiplanes as follows:

$$m_{\gamma_{i}}=\left\{\pi ,\;p_{0},\;e\right\},$$ (3)

where ***π*** represents the unit normal vector, ***p***_0_ the centroid defined by the points that compose the semiplane, and *e* the set of extrema points that limit the convex semiplane. The semiplane extraction is processed in two main steps: point candidate selection and plane fitting. In this step, the main goal is to extract three semiplanes from the input point cloud, the horizontal ground and two other arbitrary semiplanes.

**FIGURE 3:**
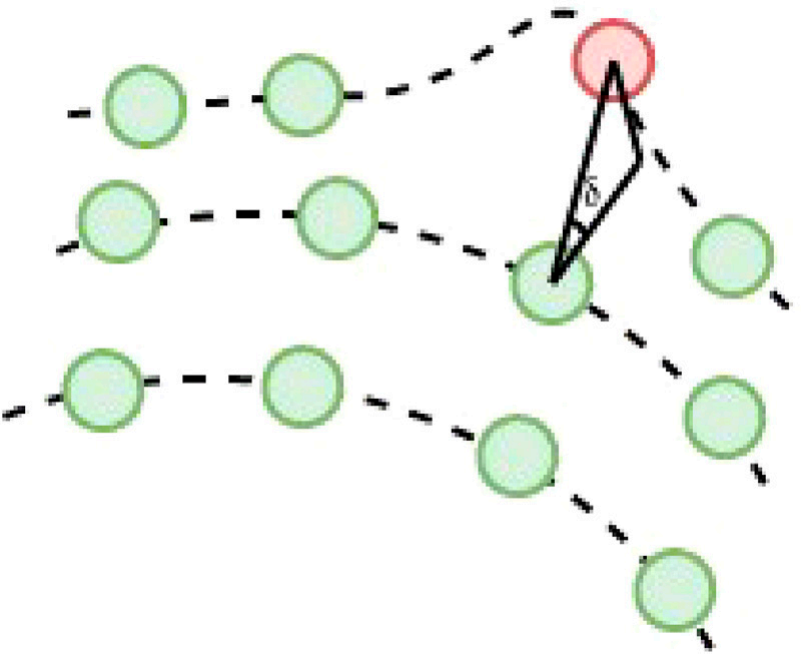
Vertical angle definition. Green dots are estimated ground points, and red dots non-ground points. The vertical angle is estimated between two consecutive points of the same column in the range image.

For selecting the ground semiplane point candidates, two different parameters are used. The first is the point vertical angle Yang et al. (2021) (Figure 3) represented as *δ*. Given the point cloud projection into the range image and two points in consecutive rows, and defining the difference between these two points as Δ ***p*** = [Δ*x*, Δ*y*, Δ*z*], the vertical angle is compute as

$$\delta =\arctan\frac{\Delta z}{\sqrt{\Delta x^{2}+\Delta y^{2}}}.$$ (4)

The second criteria is the point’s height. Given the sensor position’s prior knowledge in the robot’s referential frame, and considering that it is mounted horizontally, only points that have a height component closer to the sensor altitude are considered. Thus, if this criteria is met and if *δ* is bellow a well-defined threshold, the point is considered as candidate for the ground plane. For the two remaining planes, a simpler point candidate selection is performed. In this case, the input cloud points that were not selected as ground candidates are divided into two main sets, one on each side of the robot. With this formulation, the goal is to extract two robust semiplanes both at the right and left sides of the robot.

**FIGURE 4:**
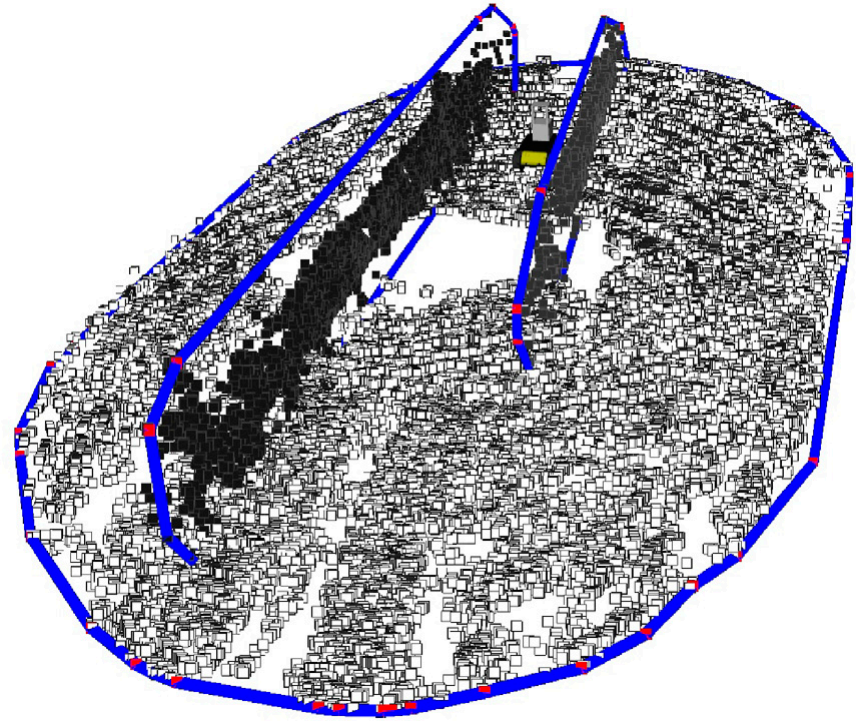
Semiplane feature extraction example in a woody crop vineyard. The blue lines represent the polygons edges, the red dots their extremas and the dark dots the semiplane inliers points.

After extracting the point candidates for the three planes, we implement a RANSAC algorithm in each set of points. This approach fits the best plane model represented by its hessian coefficients to the input set of points. In the end, the algorithm retrieves the set of inlier points that belong to the extracted plane, as well as its normal vector ***π***. This formulation outputs an infinite plane. To convert it to a semiplane, we extract a convex polygon that bounds all the inlier points that constitute the plane. To do so, a Convex Hull algorithm is applied to calculate the semiplane extremas *e*, represented in (Eq. 3). Since agricultural environments are highly unstructured, semiplane outliers can be extracted. In this work, the outliers are filtered based on the semiplane area. Only convex polygons with an area superior to a defined threshold configured by the user are considered and stored. Figure 4 shows an example of the extraction of the ground plane and the vegetation canopies in a woody crop vineyard.

### 2.2 Mapping

In the mapping stage, two registration procedures are proposed, one for points and the other for semiplanes. A 3D voxel grid is implemented and used to store the point feature map and perform efficient search algorithms. A more complex feature matching algorithm is computed, and a map merging procedure is proposed to update the semiplanes in the global map continuously. These algorithms are described in 1) and 2) respectively.

1) Point-level mapping: Unlike LeGO-LOAM’s approach that uses a standard KdTree to store feature points and map the environment, our work implements a 3D voxel grid map. This data structure, besides being less memory-efficient is more time-efficient since the searching algorithm is performed by just accessing each cell index. This 3D map is an extension of the standard 2D grid map, considering a discretization also in the *z* coordinate. Each cell is indexed by specific 3D discrete coordinates and can be efficiently accessed with these coordinates. Also, the map recognizes the different types of features supported. Thus, each cell can contain several types of features, and the searching procedures account for each type for faster processing. With this data structure, the point-based mapping procedure is performed using the information about the robot pose provided by the localization layer and using a local search algorithm. Given a set of input point features $M_{\lambda }=\left\{m_{\lambda_{1}},m_{\lambda_{2}},\ldots ,m_{\lambda_{N}}\right\}$ in their homogeneous form and the robot’s pose ***T***_*r*_, each feature is converted to the maps’ referential frame as follows:

$${\tilde{m}}_{\lambda_{i}}=T_{r}\;m_{\lambda_{i}},\;i\in \;\left\{1,\ldots ,N\right\}.$$ (5)

**FIGURE 5:**
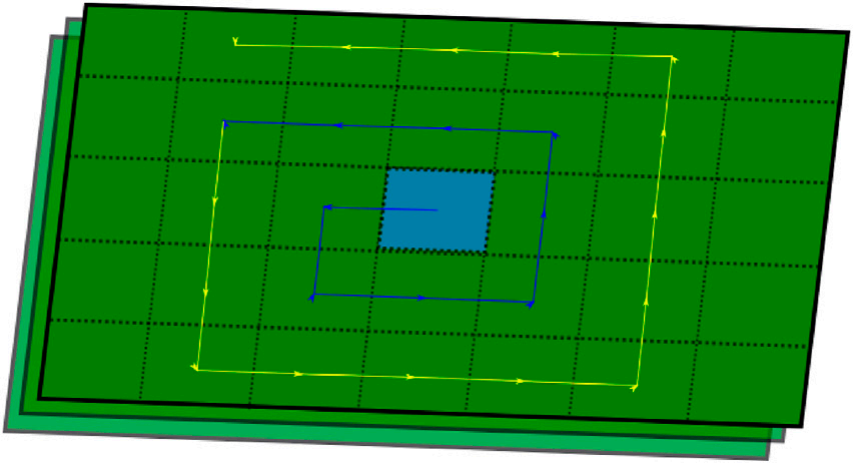
Point feature nearest neighbor local search. In a discrete 3D space, the nearest neighbor of a point feature can be in the grid map layer where the feature is located or at the top and bottom adjacent layers. A local search in these three layers is performed to find the nearest feature as described in the figure. If a feature is found when searching in the blue path, the search ends. Otherwise, the search continues through the yellow path. The user can tune the stop criteria.

Then, a local search is performed for each feature in the 3D voxel grid map. The nearest neighbor of each feature is searched by the procedure represented in Figure 5. Each feature’s nearest neighbor can be located either in its cell, in adjacent cells, or even in more distant cells. Depending on the voxel resolution, the user can specify if the local search algorithm looks for neighbors just in adjacent cells or if it continues for more remote cells in case of failure. This decision sets the stop criteria of the algorithm, that iteratively looks for the nearest feature in a region using a well-defined path as specified in Figure 5. In cases where no neighbor is found, the feature is registered and saved in the voxel map.

2) Semiplane-level mapping: The semiplane mapping procedure consists of two main tasks: semiplane matching and registering. The first step is to start with the matching algorithm, like in the point features case, to convert the observed semiplanes to the maps’ referential frame. Thus, we apply a similar transformation as the one represented in (Eq. 5) to the semiplane inlier points and extremas. Then, to improve the matching procedure, we refine the normal vector estimation of the transformed semiplanes using a Principal Component Analysis (PCA) algorithm. Given the set of transformed semiplane points ${\tilde{M}}_{\gamma }=\left\{{\tilde{m}}_{\gamma_{1}},{\tilde{m}}_{\gamma_{2}},\ldots ,{\tilde{m}}_{\gamma_{N}}\right\}$ , we define ***S*** = ***YY*** ^*T*^, where

$$Y=\left[\begin{array}{c} {\tilde{m}}_{\gamma_{1}}-\tilde{p_{0}}\\ {\tilde{m}}_{\gamma_{2}}-\tilde{p_{0}}\\ \ldots \\ {\tilde{m}}_{\gamma_{N}}-\tilde{p_{0}} \end{array}\right]$$ (6)

and $\tilde{p_{0}}$ represents the transformed semiplane centroid. The refined normal vector corresponds to the eigenvector of ***S*** with the smallest eigenvalue. After this, the matching procedure is computed considering three different correspondence criteria:

• Overlapping area;
• Normal vector difference;
• Centroid-to-plane distance.

If an observed and a global map’s semiplane meet these criteria, one can conclude that both planes overlap, have the same orientation, and are at the same position. Thus, they are considered as correspondences.

To compute the overlapping area between semiplanes, the local plane reference frame is extracted from the normal vector, and the semiplanes boundaries are projected into this frame. The *z* component is ignored since it is expected that, in the local plane reference frame, the boundaries lie in the X-Y plane (*z* = 0). Working in two dimensions, the interception between the two polygons is computed, and its area is calculated. If the overlapping area is higher than a threshold level, the normal vector difference is computed to check if the planes have the same orientation. Finally, the distance between the observed semiplane centroid to the semiplane in the global map is computed.

After the matching step, semiplane registration is performed to build the semiplane global map iteratively. Semiplanes that were not matched with any in the global map are directly registered in the global map. For the ones that were matched, a map merging algorithm is proposed. The semiplane representation is recomputed by merging inliers points between the two correspondences, recalculation of the normal vector using (Eq. 6), and boundary merging using Convex Hull. This process allows semiplanes to grow with newly observed features and to map large bounded surfaces. The process of registration can be observed in the following video: https://youtu.be/Yx8el67eTCw.

### 2.3 Localization

The localization procedure aims to compute the robots’ 6-DoF pose using the feature extraction and mapping algorithms described. To this purpose, in contrast with the state-of-the-art, this work implements a PF with the novelty of considering point-semiplane particle weight calculation. This means that the filter can consider and balance both feature modalities and work in the absence of each one of them. The PF is standardly divided into three main steps: a prediction step where the particles are innovated through a motion model, the particles weight calculation given the observed features, and the resampling step to replace particles with lower weight by others with higher weight. These three steps are described in 1), 2) and 3) respectively.

1) Motion model: For predicting the particles likelihood distribution, they are innovated through a 6-DoF model proportional to an estimated relative motion. A LiDAR odometry algorithm based on the Iterative Closest Point (ICP) Besl and McKay (1992) method is implemented to estimate the frame-to-frame 6-DoF robot motion, where an input wheel odometry control ***u***_*t*_ is used as first guess for the iterative algorithm. The particles spreading is proportional to the distance measured by the scan-matching algorithm *d*_*u*_. Let us define the following matrices:

• Δ ***T***: the LiDAR odometry increment represented as an homogeneous transformation; and
• ***T***_*n*_: an homogeneous transformation matrix computed by sampling a minimal parameter space (6-DoF) from a Gaussian distribution with standard deviation *d*_*u*_.

Thus, a particle *j* is innovated at the instante *t* as follows:

$$T_{j,t}=T_{j,t-1}\;\Delta T\;T_{n}.$$ (7)

2) Weight update: The particles weight calculation is the most critical and innovative step of the proposed localization procedure. The major challenges are the consideration of multi modal inputs (points and semiplanes) and the creteria balance on the semiplane-based weight calculation, since two parameters are used to compute the weight. To account for both modalities, a two-step weight calculation is performed using two subfunctions: *W*_*λ*_ for the point (edge and planar) features and *W*_*γ*_ for the semiplane features. The particles weight is represented as a likelihood function *P*(***z***_*t*_| ***T***_*j*,*t*_, *Z*_0:*t*−1_) where ***z***_*t*_ represent the feature observations at the instant *t*, ***T***_*j*,*t*_ the particle’s pose, and *Z*_0:*t*−1_ the map build so far.

In the point-feature case, features are converted to maps’ reference frame using each particle pose as follows:

$${\tilde{m}}_{\lambda_{i}}=T_{j,t}\;m_{\lambda_{i}},\;i\in \;\left\{1,\ldots ,N\right\}.$$ (8)

Then, correspondences are found using the 3D voxel grid map search algorithms described in Section 2.2. Considering the set of *K* correspondences found $\left\{{\tilde{m}}_{\lambda_{i}}↔m_{\lambda ,g_{i}}\;:\;i\in \left\{1,\ldots ,K\right\}\right\}$ , where the subscript *g* denotes for features in the *global* map, the point-feature weight subfunction is computed as

$$W_{\lambda }=\frac{1}{\sqrt{2\pi }\sigma_{\lambda }}\overset{K}{\underset{i=1}{\sum }}\exp\left(\frac{-1}{\sigma_{\lambda }}\cdot \|{\tilde{m}}_{\lambda_{i}}-{\tilde{m}}_{\lambda ,g_{i}}\|\right),$$ (9)

where *σ*_*λ*_ is the standard deviation of the point-feature measurement, and ‖.‖ represents the L2 norm. This formulation states that the weight of the particle increases exponentially with the decrease of distance between two correspondences, and that it is as higher as the number of correspondences *K* found.

In the semiplane-feature case, the first step is also the conversion of them to the maps’ reference frame. The set of extremas of each semiplane *e*_*i*_ and its corresponding centroid $p_{0_{i}}$ are converted to this referential using each particle pose as in (Eq. 8). Then, correspondences between the observed semiplanes and the ones already registered in the global map are searched using the three criterias described in Section 2.2: overlapping area, normal vector difference, and centroid-to-plane distance. Given the set of *K* correspondences found $\left\{{\tilde{m}}_{\gamma }↔m_{\gamma ,g_{i}}\;:\;i\in \left\{1,\ldots ,K\right\}\right\}$ , where the subscript *g* denotes for features in the *global* map, the semi-plane weight subfunction is modelled as a multivariable function as follows:

$$W_{\gamma }=\overset{K}{\underset{i=0}{\sum }}w_{\gamma }\left(\pi_{i}\right)\cdot w_{\gamma }\left(p_{0_{i}}\right),$$ (10)

where

$$\begin{array}{ll} w_{\gamma }\left(\pi_{i}\right) & =\frac{1}{\sqrt{2\pi }\sigma_{\pi }}\exp\left(\frac{-1}{\sigma_{\pi }}\cdot \|{\tilde{\pi }}_{i}-{\tilde{\pi }}_{g_{i}}\|\right)\\ w_{\gamma }\left(p_{0_{i}}\right) & =\frac{1}{\sqrt{2\pi }\sigma_{p_{0}}}\exp\left(\frac{-1}{\sigma_{p_{0}}}\cdot D\left(p_{0_{i}},{\tilde{m}}_{\gamma ,g_{i}}\right)\right), \end{array}$$

**FIGURE 6:**
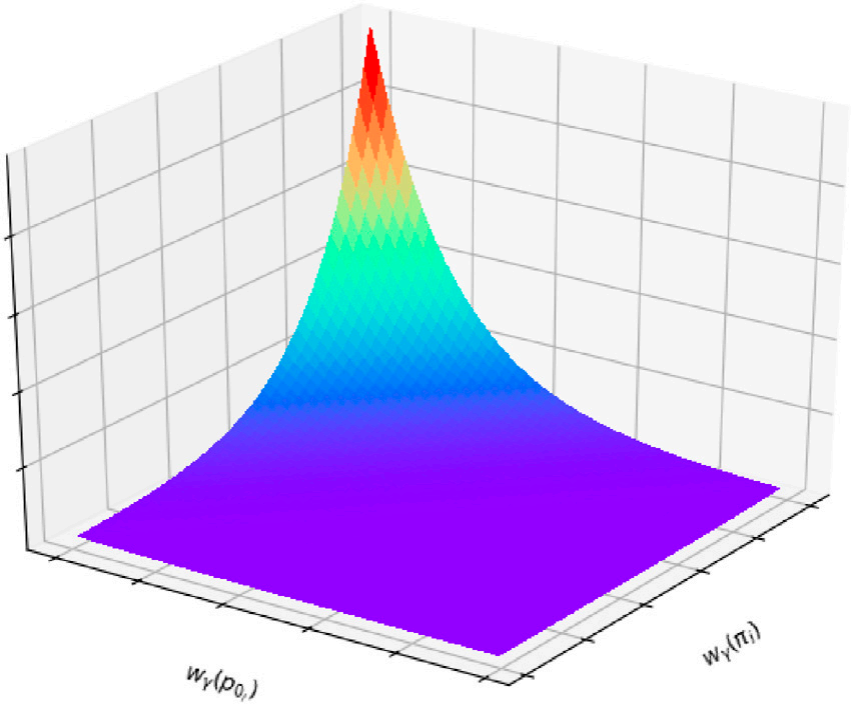
Likelihood of the semiplane-based weight calculation represented as a multivariable function. The likelihood decreases exponentially with the increase of difference between normal vectors and centroid-to-plane distance. Their corresponding standard deviations can control the impact of each one of the variables in the final likelihood.

*σ*_***π***_ and $\sigma_{p_{0}}$ represent the standard deviations of the normal vector and centroid measurements respectivelly, ${\tilde{\pi }}_{\left(.\right)}$ the semiplane’s normal vector, ${\tilde{p}}_{0_{\left(.\right)}}$ the semiplane’s centroid, and *D*(.) the point-to-plane distance operator. Figure 6 represents the semiplane-based weight for a single correspondence. As represented, the importance given to the normal vector difference (to account for plane rotation) and to the centroid-to-plane distance (to account for plane displacement) can be tuned by the standard deviations of each measurement. The tunning step can be challenging since the multivariable function considers two variables in different spaces (vectors and distances). Overall, the particle’s weight decreases exponentially with the increase of difference between correspondence’s normal vector and centroid-to-plane distance.

Given the definitions (Eqs 9, 10) the particle likelihood is computed as

$$P\left(z_{t}|T_{j,t},Z_{0:t-1}\right)=W_{\lambda }\cdot W_{\gamma }.$$ (11)

In this way, the particle with the highest weight is the one that presents the best alignment both in the point-feature and semiplane-feature spaces. The final robot pose per frame is computed by the weighted average of all particles’ pose.

3) Resample: The resampling step of the PF is used to substitute low-weight by high-weight particles. In this work, the multinomial resample algorithm Douc and Cappe (2005) was implemented to accomplish this. This approach draws *N* samples from a uniform distribution *u*_*i*_ and selects the particle *j* for replication if

$$u_{i}\in \left[\overset{j-1}{\underset{p=1}{\sum }}w_{p},\overset{j}{\underset{p=1}{\sum }}w_{p}\right),$$ (12)

where *w*_*p*_ represents the particle’s *p* weight. To avoid the well-known problem of particle degeneracy that happens when either all the particles are in the wrong place or they are highly condensed, resampling is not executed for all iterations. This method is only employed when a significant robot motion is observed (either in translation or rotation in the six degrees of freedom). The user can set the amount of motion required to perform resampling.

## 3 Simulation Experiments

This work’s major innovation is the use of semiplanes to map the environment and localize the robot within it. As discussed in Section 2.3, one of the biggest challenges in the proposed semiplane-based localization is the weight given to the normal vector difference and the centroid-to-plane distance. This Section presents two simulation experiences to validate the numeric stability of the approach. Three orthogonal planes are used to localize the robot in the simulated environment and no point-features are considered.

### 3.1 Methodology

**FIGURE 7:**
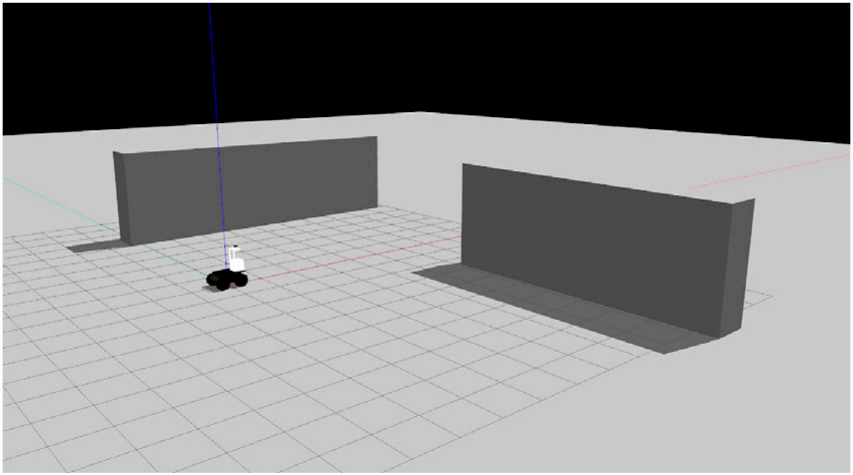
Simulation environment containing three perpendicular planes: the ground and two walls.

The simulation’s main goal is to verify if, with three orthogonal planes is possible to localize the robot. Thus, the environment present in Figure 7 was built containing two perpendicular walls. The third semiplane is the ground. The robotic platform used for real experiments that will be detailed in Section 4 was modeled and inserted in the simulation. This platform is based on a Husky robotic base. To obtain the raw wheel odometry inputs, the Husky simulator^1^ was used, that considers the error caused by the wheel slippage. To obtain the LiDAR data, the Velodyne simulator^2^ was used with a simulated Gaussian noise with standard deviation of 0.008 m.

Given all of the above, the simulation experiments were carried out by two different sequences: a translation-only motion and a rotation-only motion. The idea is to validate that, using only three perpendicular semiplanes, the proposed approach can estimate translations and rotations. The PF algorithm used 500 particles to estimate the robot’s motion.

### 3.2 Localization Performance

**FIGURE 8:**
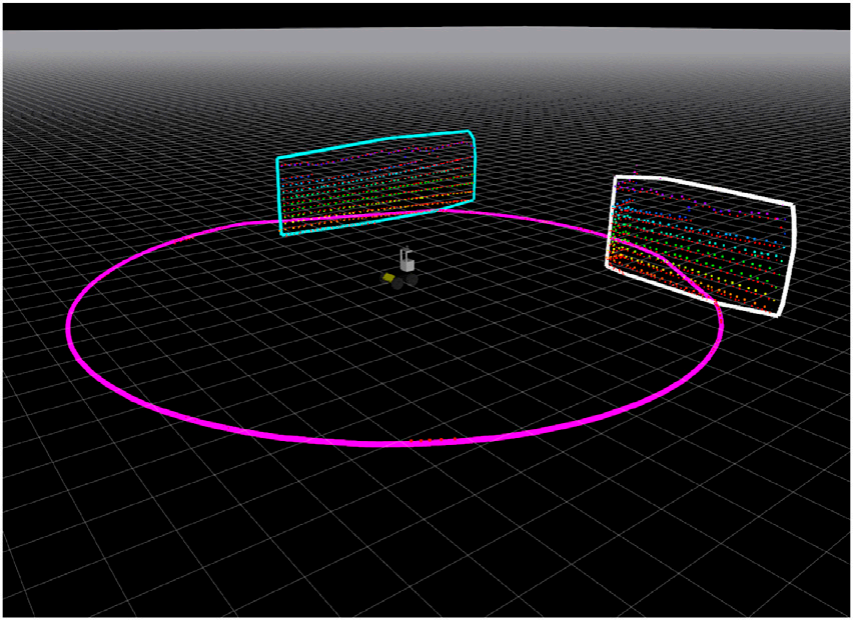
Semiplane feature extraction of the three simulated semiplanes.

**FIGURE 9:**
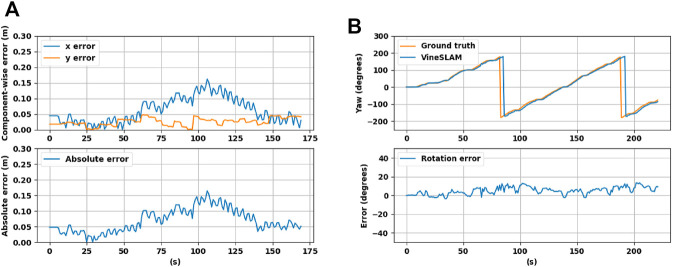
Simulation results using three perpendicular planes in the localization and mapping procedures for a **(A)** translation-only trajectory, and for a **(B)** rotation-only trajectory.

As referenced before, under the same simulated environment, two different experiments were performed. The semiplane feature extraction procedure describe in Section 2.2 resulted in the successful detection of the three semiplanes (two walls and the ground) as shown in Figure 8. In the first experiment, the robot moves forward without rotating. Figure 9A shows the *x* and *y* deviation to the ground truth, as well as the absolute distance error (meters). Overall, the semiplane-based localization procedure presents an average distance error of 0.069 1 m for this sequence. This shows that the semiplane extraction algorithm is accurate in estimating the normal vectors, centroids and extremas. More importantly, using only three orthogonal semiplanes, the localization procedure can localize the robot with low error.

Regarding the rotation-only experiment, Figure 9B shows the estimated yaw rotation in reference with the ground truth, as well as the corresponding rotation error. For this sequence, the average rotation error obtained was 5.01 degrees. One of the well-known localization issues is the estimation of motion in rotation-only movements. These scenarios are more challenging than translation motions since the perception of the environment changes faster, challenging the matching procedures. Besides, the odometers tend to present higher errors in rotations due to wheel slippage. For all these reasons, this type of motion shows to impact the filter’s performance. Even so, the localization approach can estimate the robot rotation with acceptable performance, using only three semiplanes.

In the real world, the presence of at least three orthogonal semiplanes is not always guaranteed. For example, in vineyards, the most common scenario is detecting the ground plane and two parallel vegetation planes. With this configuration there are no constraints to estimate the forward component of the translation. Due to this, the proposed approach fuses points with semiplanes. Nevertheless, this simulation aims to show that the proposed formulation is suitable for semiplane-only localization. The simulated experiments performed show that the algorithm can perform accurately in translational and rotational movements.

## 4 Results

**FIGURE 10:**
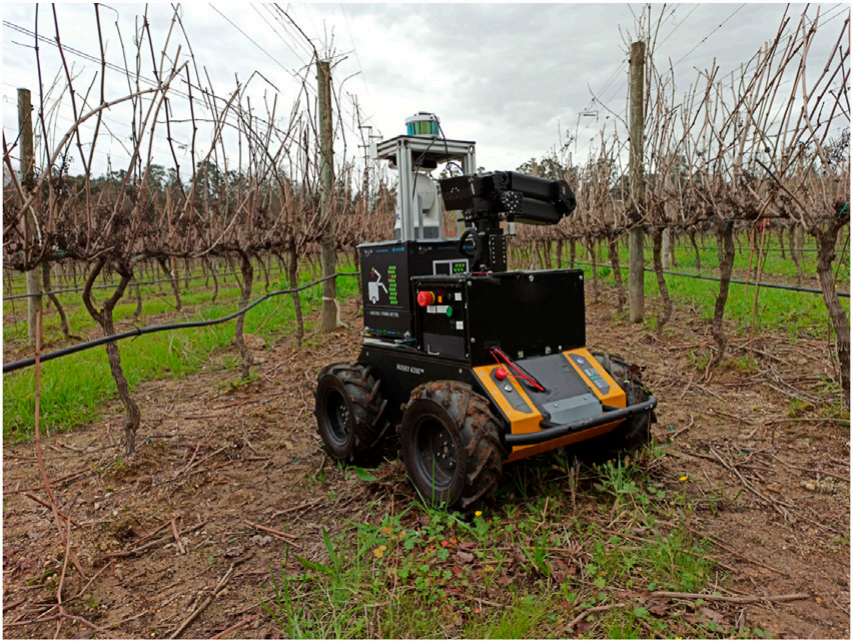
AgRob V16 robotic platform used to test the proposed approach placed in a woody-crop vineyard.

To test the proposed solution, our robotic platform AgRob V16 Aguiar et al., 2020b; Santos et al. (2020) present on Figure 10 was used. The robot is equiped with a Velodyne Puck (VLP-16) and was placed in Aveleda’s vineyard, in Portugal. It travelled three different sequences that will be described later on. Section 4.1 details the experiments performed in this context, and Section 4.2 describes and analysis the results obtained.

### 4.1 Methodology

**FIGURE 11:**
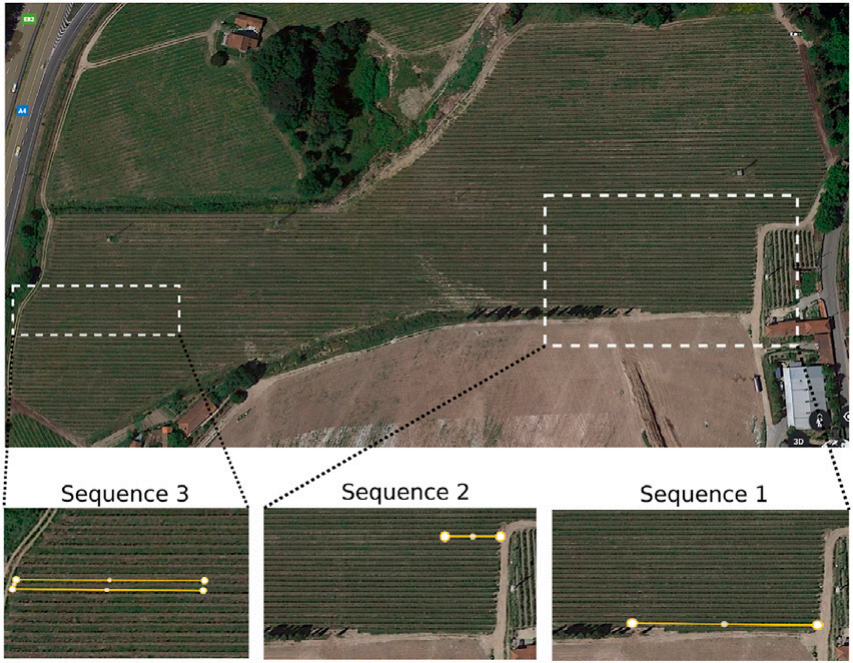
Satellite image of Aveleda’s vineyard. The sub-figures represent the sequences (1, 2 and 3) traveled by the robot.

The proposed VineSLAM localization and mapping algorithm performance was analysed in three different sequences described in Table 2. The characteristics of each sequence are different not only due to the different travelled paths, but also because they were recorded in different seasons of the year. Two of them in the summer, which means that the vineyard present a high density of foliage, and other in the winter and without foliage. Sequence 1 has almost 70 meters of extension and is the less symmetric sequence since, besides being inside a corridor, it is at the border of the vineyard. Thus, the high-range LiDAR used can capture scene objects that are not present in the corridor. Sequence 2 presents the smallest path (23.52 meters). Nevertheless, it is challenging since the path is inserted in the middle of the vineyard, with a high density of corridors. This sequence was recorded during the winter, which means that the vineyard had not foliage. Finally, sequence 3 is the most extensive, with more than 80 meters. In this, the robot travels along two vineyard corridors and is also placed in the middle of the vineyard. Thus, in most cases, the environment is highly symmetric, compromising the localization and mapping algorithm. Figure 11 shows a satellite image with the three sequences represented.

**TABLE 2:** Summary of the experiments performed in Aveleda’s vineyard.

Experiment	Distance (m)	Foliage stage	Season
Sequence 1	69.73	With	Summer
Sequence 2	23.52	Without	Winter
Sequence 3	81.72	With	Summer

To test and our approach, the proposed VineSLAM algorithm was executed in the three sequences and compared with the state-of-the-art LeGO-LOAM (Shan and Englot, 2018) SLAM method. We consider LeGO-LOAM the state-of-the-art in outdoor 3D SLAM using LiDAR sensors. This method was tested in extensive outdoor experiments, and proved to perform better than its ancestor, LOAM. From the literature, one can see that LeGO-LOAM is aligned with robust 3D SLAM approaches, such as G-ICP (Ren et al., 2019). Thus, in this work we test LeGO-LOAM in harsh agricultural environments, and benchmark it against our approach, VineSLAM.

To validate the results, Global Navigation Satellite System (GNSS) was used as ground truth, and the Absolute Pose Error (APE) was measured using this reference. The maximum, mean and Root Mean Squared (RMS) APE errors were annotated for each experiment.

### 4.2 Localization Performance

The robot localization estimation is evaluated in sequences 1, 2 and 3 and compared with the state-of-the-art SLAM approach LeGO-LOAM. It is worth noting that the sequences are present in long vineyard corridors, which can be problematic for SLAM approaches. One of the key issues of SLAM is the well-known corridor problem where the forward component of motion has high uncertainty due to the symmetries of the scene. In the Aveleda vineyard, this can happen since vine trunks are equally spaced, and consequently, the agricultural environment is highly symmetric.

**FIGURE 12:**
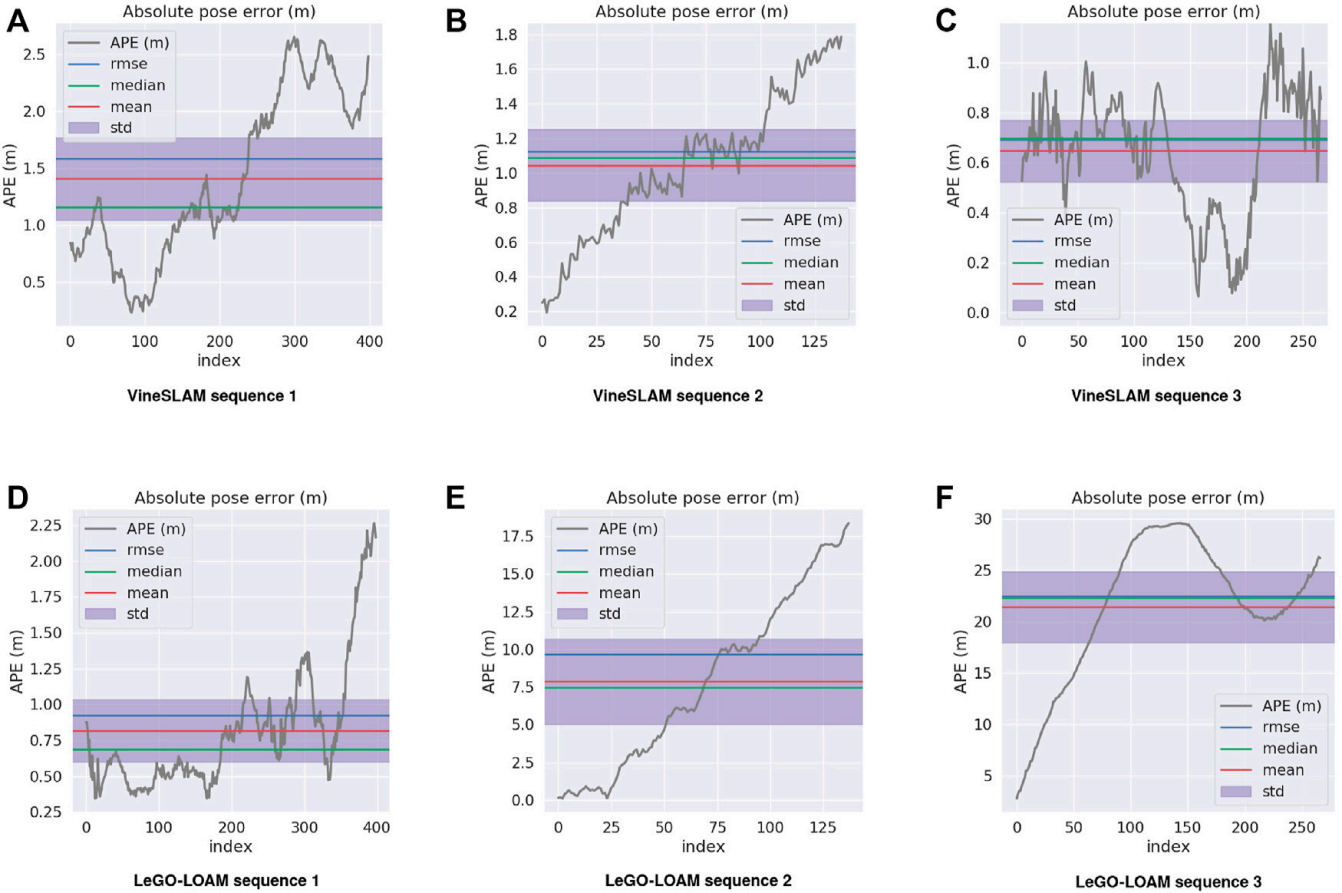
Average pose error (m) and its corresponding Root Mean Squared Error (RMSE), median, mean and standard deviation for **(A–C)** VineSLAM and **(D–F)** LeGO-LOAM under the three experiments performed.

**FIGURE 13:**
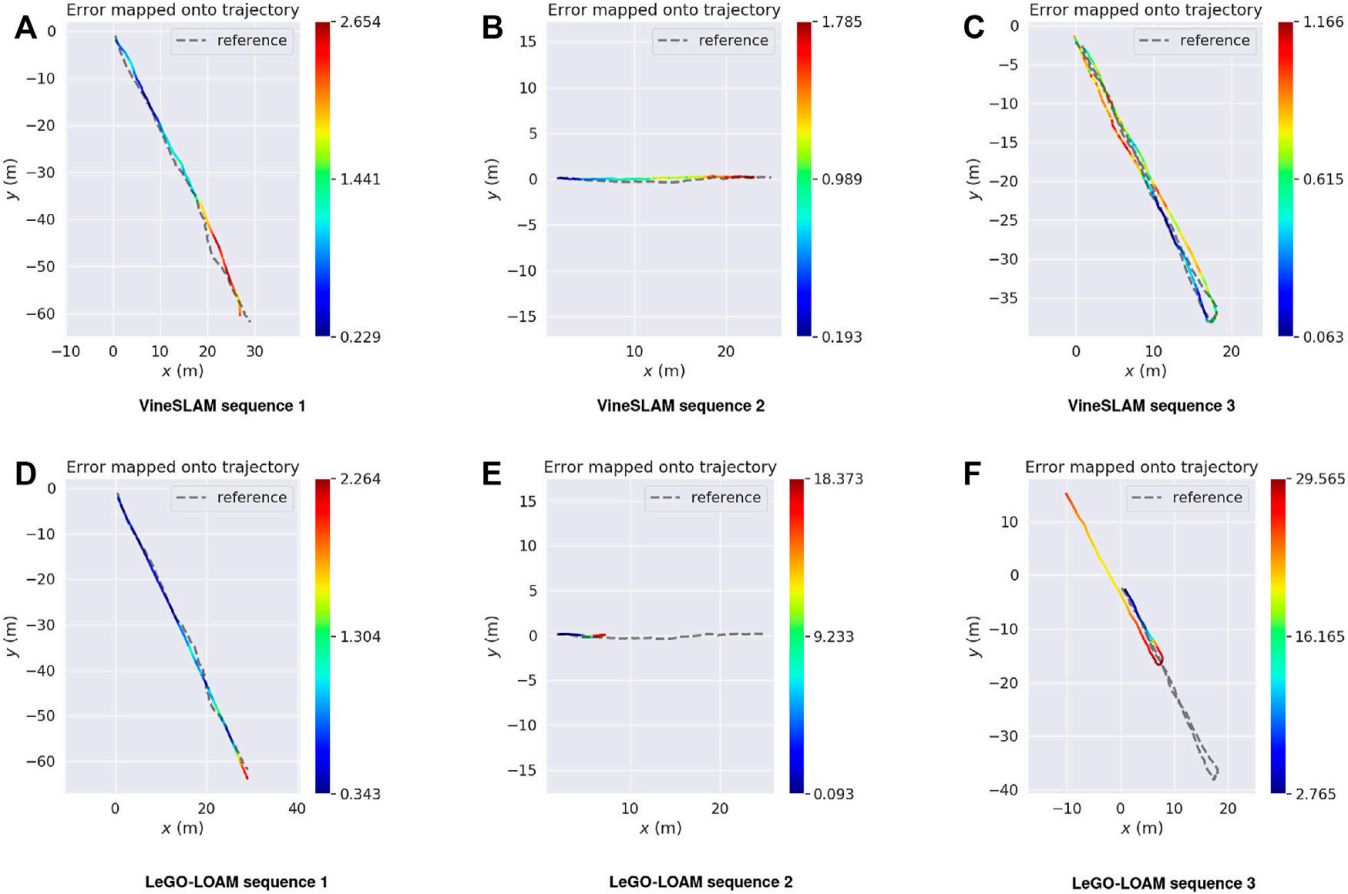
Absolute pose error (m) mapped onto the trajectory for **(A–C)** VineSLAM and **(D–F)** LeGO-LOAM with reference to the ground truth.

**FIGURE 14:**
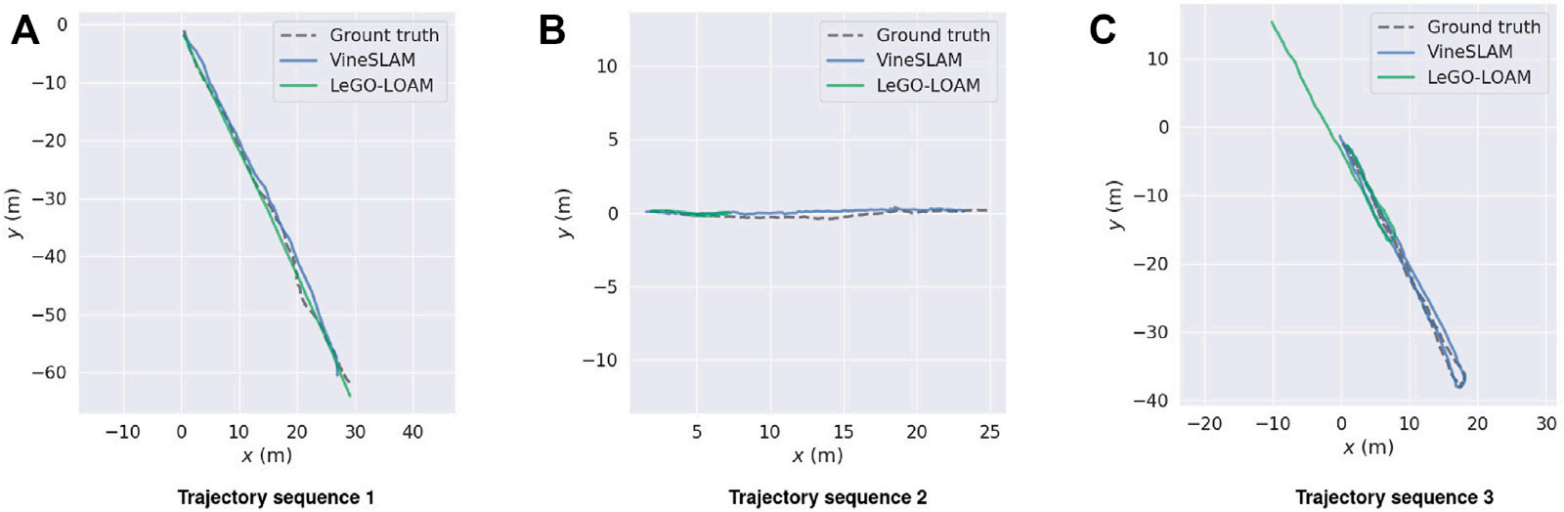
VineSLAM’s and LeGO-LOAM’s localization estimation with reference to the ground truth for sequences **(A)** 1, **(B)** 2, and **(C)**3.

The APE is used to analyze the obtained results. For each timestamp, the absolute difference between the reference and the estimated poses is calculated. Table 3 summarizes the results obtained for both methods in the three sequences. Figure 12 plots the APE over time, as well as its corresponding RMS error, mean, median and standard deviation. To highlight the APE during the robot motion, Figure 13 maps it onto the trajectory and represents the error through a color code. Finally, to have a clear perception of our VineSLAM approach and LeGO-LOAM in reference with the ground truth, Figure 14 presents all the trajectories in the same graphic for each sequence.

**TABLE 3:** Absolute pose error metrics for VineSLAM and LeGO-LOAM under the three test sequences.

Experiment	Method	Max (m)	Mean (m)	RMS (m)
Sequence 1	VineSLAM	2.65	1.41	1.58
	LeGO-LOAM	2.26	0.81	0.93
Sequence 2	VineSLAM	0.84	0.38	0.44
	LeGO-LOAM	20.81	10.49	11.87
Sequence 3	VineSLAM	1.17	0.64	0.69
	LeGO-LOAM	29.57	21.39	22.48

For sequence 1 it is possible to verify that LeGO-LOAM performs better than VineSLAM. In particular, for this sequence, the state-of-the-art approach outperforms VineSLAM in approximately 0.6 meters considering the RMS error. This was the sequence where LeGO-LOAM presented better performance since, as referenced before, it could find structure outside the corridor placed in the vineyard’s border. As can be observed in Figure 13A VineSLAM present the higher error when the robot performs a rotation inside the corridor. Even so, both methods showed acceptable performance even in this challenging environment.

For sequences 2 and 3, we verified that LeGO-LOAM localization estimation has degenerated, i.e., the state-of-the-art algorithm fails to estimate the robot pose for these two sequences. The environment’s high symmetry impacts its localization algorithm since, in many instants, LeGO-LOAM estimates that the robot is stopped when it is moving. Thus, for sequence 2 this method presents an RMS error of 11.87 meters and for sequence 3 22.48 meters. Figure 12E shows that LeGO-LOAM accumulates error over time for sequence 2. For the other sequence, the same happens until the robot turns around, the moment where this method starts estimating movement again. In these two sequences, the proposed VineSLAM approach’s contribution is highlighted since it can maintain a precise robot localization in both of them. Especially for sequence 3, where the robot travels more than 80 meters over two vineyard corridors, VineSLAM achieved an RMS error of 0.69 meters.

Overall results show that VineSLAM is suitable for localization and mapping in agricultural environments. The PF approach considers not only point-features but also semiplanes to map and localize the robot. This approach proved to be accurate even in challenging agricultural environments, improving the state-of-the-art. In most cases, LeGO-LOAM underestimates translation due to the corridor’s symmetry, while the PF approach proposed in VineSLAM can overcome this issue using a discretization of the 6-DoF state space in 500 different particles. From Figure 14 one can verify that VineSLAM follows the GNSS reference with accuracy in the three sequences while LeGO-LOAM only does so in the first. This proved that the localization and mapping research is still open for improvement. For harsh environments such as vineyards, dedicated approaches should be proposed to tackle the more generic state-of-the-art algorithms’ limitations.

## 5 Conclusion

This work proposes an extension of the state-of-the-art in localization and mapping oriented to agricultural robots. In this context, we propose VineSLAM, an algorithm that uses both points and semiplanes to map the environment and localize agricultural robots. The integration of all this information in a single pipeline is done efficiently with a 3D voxel map proposal to accelerate search algorithms and innovative semiplane-based mapping techniques. Also, a PF is used with a novel update step, where the likelihood of the particle considers both feature modalities. Results show that our formulation can localize a robot using only three orthogonal semiplanes. Under real-world experiments in a woody-crop vineyard, VineSLAM achieved RMS errors of 1.58, 0.44, and 0.69 meters for three sequences. Overall, our approach outperforms the state-of-the-art LeGO-LOAM algorithm that fails in two of the three sequences.

In future work, we would like to extend the mapping algorithms of VineSLAM. In particular, features with semantic representations will be extracted from agriculture environments, such as trunks and fruits, and used in the mapping and localization procedures. Additionally, we would like to partition the global map in a graph-like fashion considering a topological structure. A sensor fusion approach will be adopted to improve the localization redundancy and robustness. Finally, the algorithm will be tested in different agricultural scenarios such as greenhouses and orchards.

## Data Availability

The original contributions presented in the study are included in the article/Supplementary Material, further inquiries can be directed to the corresponding author.

## Footnotes

1
https://github.com/husky/husky_simulator

2
https://bitbucket.org/DataspeedInc/velodyne_simulator.git
